# Investigating serum iodine nutritional levels and their correlation with thyroid function in adult patients with Graves’ hyperthyroidism at different treatment stages

**DOI:** 10.3389/fendo.2025.1628670

**Published:** 2025-08-27

**Authors:** Lilan Wang, Zixuan Ru, Shengnan Gao, Na Lv, Kerou Li, Hong Qiao

**Affiliations:** Department of Endocrinology, Second Affiliated Hospital of Harbin Medical University, Harbin, China

**Keywords:** Graves’ disease hyperthyroidism, iodine nutritional status, serum iodine concentration, thyroid function, precise iodine supplementation

## Abstract

**Objective:**

Investigate the thyroid function and thyroid autoantibodies in adult Graves’ disease (GD) patients at different treatment stages and healthy adults with serum iodine concentration (SIC), and analyze the relationship between SIC and thyroid function as well as thyroid autoantibodies.

**Method:**

GD-induced hyperthyroid patients and healthy normal controls will be recruited from the Endocrinology Clinic. Peripheral venous blood samples will be collected, and the SIC content will be determined by the arsenic cerium catalytic spectrophotometry method. Thyroid function indicators will be detected by electrochemical immunoassay analysis, the Pearson/Spearman correlation analysis will be used to analyze the correlation between SIC and thyroid function indicators.

**Results:**

A total of 510 participants were included, of which 80 were healthy adults and 430 had GD. The SIC levels were 63.78 ± 18.92μg/L and 74.09 ± 29.85μg/L. There was a significant difference in SIC levels (100.15 ± 40.25μg/L vs 65.74 ± 19.37μg/L, P < 0.05). In terms of iodine deficiency degree (<45μg/L), there was no significant difference between the two groups (11.25% vs. 12.50%, P>0.05). Correlation analysis showed that SIC was positively correlated with FT4 and TRAb (r=0.213, r=0.369, P<0.05). In newly diagnosed GD patients, the TPOAb concentration and positivity rate in the low blood iodine group were higher than those in the high blood iodine and appropriate blood iodine groups (P<0.05). The TgAb level in the high blood iodine group was greater than that in the low blood iodine and appropriate blood iodine groups (P<0.05), while the TgAb positivity rate in the low blood iodine group was higher than that in the high blood iodine and appropriate blood iodine groups, although there was no significant difference (P>0.05).

**Conclusions:**

1.The SIC levels in GD patients are higher than those in the healthy control group, and the SIC levels in untreated GD patients are higher than those in treated GD patients.2.There is a potential association between SIC and thyroid function indicators. In newly diagnosed GD patients, SIC shows a positive correlation trend with FT4 and TRAb.3.There are differences in the distribution of patient groups with different iodine contents in the blood, and there are also differences in the levels and positivity rates of TPOAb and TgAb.

## 1 Introduction

Graves’ disease (GD) is an autoimmune thyroid disorder and the most common cause of hyperthyroidism. Its occurrence is caused by various factors, among which genetic factors account for about 79%, and environmental factors account for about 21%. Among the environmental factors, iodine plays a crucial role in the synthesis of thyroid hormones and the maintenance of thyroid hormone homeostasis, and has a key role in the occurrence and development of the disease (1). Extensive research confirms that chronic iodine deficiency or excess disrupts the iodine nutritional balance, leading to abnormal serum iodine concentration (SIC), and there is an U-shaped relationship between SIC and the incidence of thyroid diseases, meaning that the incidence of thyroid diseases increases both when iodine intake is too high and too low (2).

Since 1996, China has implemented the Universal Salt Iodization (USI) policy, covering all 31 provinces, including Heilongjiang, leading to the gradual improvement of the iodine nutritional status of the population (3). But in recent years, the incidence of thyroid diseases has increased, and people’s health awareness has also improved, so now people are increasingly concerned about their iodine nutritional status and intake, especially for patients with autoimmune thyroid diseases such as GD and hyperthyroidism (4). To achieve personalized iodine nutritional balance for GD and hyperthyroidism patients and comply with China’s policy of “regional differentiated approach, categorized guidance, personalized intervention, and scientifically precise iodine supplementation,” it is first necessary to accurately determine the current iodine nutritional level of GD and hyperthyroidism patients in different regions.

Currently, there is no universally recognized method for evaluating individual iodine nutrition, and the evaluation of iodine nutrition in the population mainly focuses on dietary iodine intake or urinary iodine (UI) content. When evaluating individuals, indicators such as thyroid volume (Tvol) and SIC are used. However, the dietary iodine intake thresholds recommended by the guidelines do not take into account the issue of iodine imbalance in patients with thyroid diseases. The urinary iodine levels (UI) show significant variation due to seasonal changes, sampling time, and the iodine content in food and water sources. Random measurements of UI do not reflect an individual’s iodine status (5). Although the 24-hour urinary iodine excretion (24h-UIE) can accurately reflect daily iodine excretion, its application is limited due to the difficulty in completely collecting 24-hour urine (6). The TVol is often used to examine iodine status over a long period, but it is not suitable for immediate monitoring. Recent studies have shown that iodine in the blood mostly exists in the form of iodine-bound thyroid hormones and iodide ions, which are closely related to the bioavailable iodine supply available for thyroid utilization.

The SIC measurement reflects the true iodine content in the human body, is more stable than UI, has less individual variation, and is a biomarker for accurately evaluating short-term iodine nutritional status. For patients with GD and hyperthyroidism, simultaneously measuring thyroid function and SIC can avoid the inaccuracy of random UI testing and the inconvenience of 24-hour UIE collection. Studies show that in adults, SIC is significantly positively correlated with UIC (r>0.65, P<0.01), and SIC is relatively stable, unaffected by age or gender. This study proposes that SIC can be used as an indicator of iodine status (7). Therefore, SIC provides a more convenient, rapid, and accurate method for personalized iodine status assessment for patients with GD and hyperthyroidism. Establishing the medical reference range for SIC will form a new iodine nutrition evaluation system for SIC and can promote precise iodine intervention measures for GD patients.

**Figure 1:**
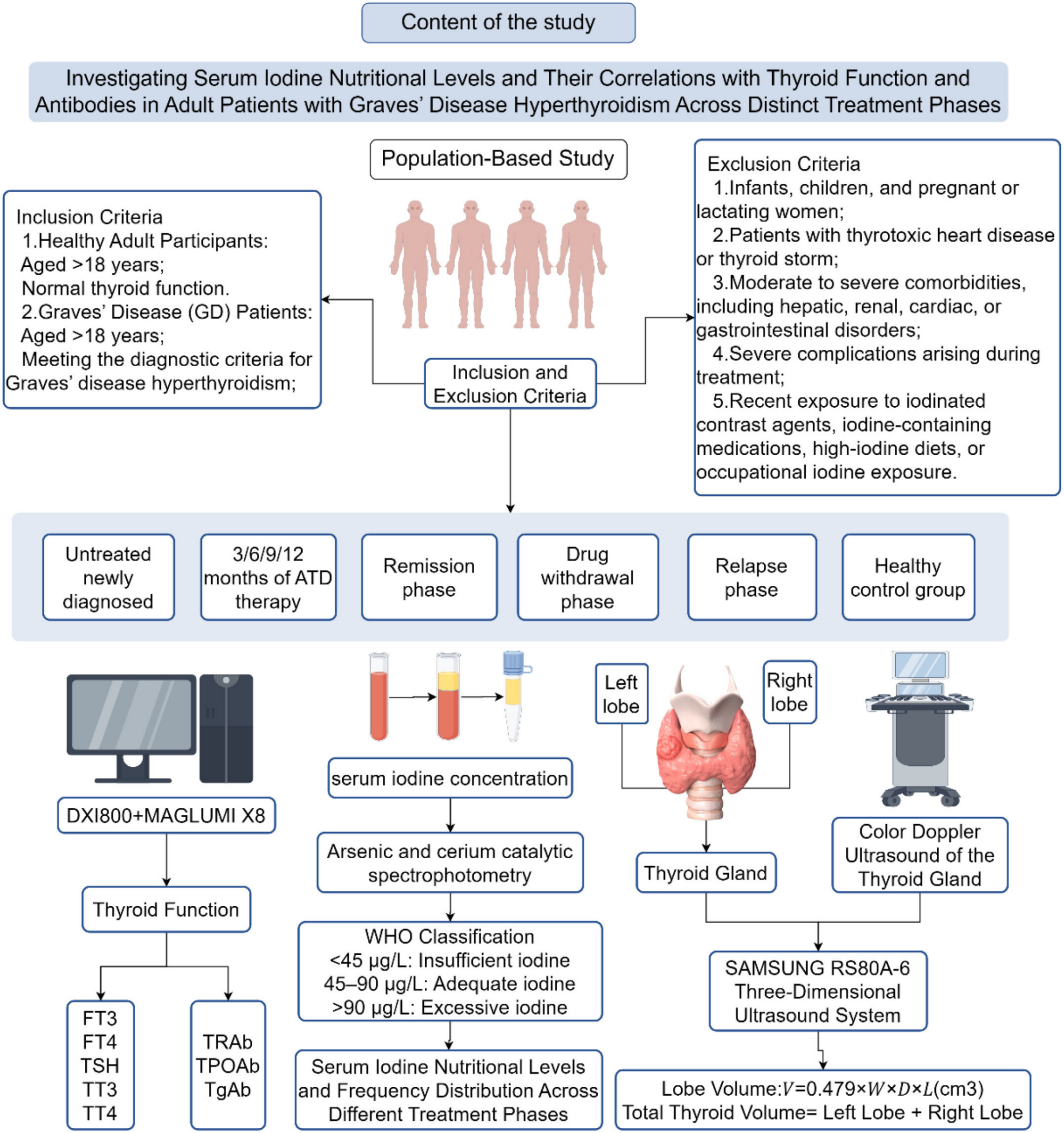
Flowchart of the study progress.

Currently, there is a lack of research on SIC reference ranges for GD patients at home and abroad. Well-known international laboratories such as the Mayo Clinic, Quest Diagnostics, and the WHO have all provided reference ranges, but they have not distinguished them for different populations. In China, the country is also creating standardized SIC reference ranges and considers personalized standards as a key research direction (8). his study will examine the SIC content levels in adult GD patients at different treatment stages (initial diagnosis, drug therapy, discontinuation period, recovery, and recurrence) as well as in adults with normal thyroid function, to provide reference data to determine the SIC ranges for various populations that comply with China’s USI policy. And we also explored the correlation between SIC and thyroid function parameters. By adopting the SIC reference range specified by the WHO, we applied the distribution frequency of iodine status (excessive, sufficient, deficient) and the positive rate of TPOAb/TgAb to newly diagnosed GD patients without treatment. These findings can provide a basis for personalized iodine intervention at different stages of GD treatment, thereby supporting a strategy of scientifically supplementing iodine guided by classification to treat thyroid disease patients (**Figure 1**).

## 2 Study participants and methods

### 2.1 Study participants

The study will enroll eligible participants between September 2024 and March 2025 at the Endocrinology outpatient clinic of Harbin Medical University’s Second Affiliated Hospital. Two distinct groups will be formed: adult patients with confirmed GD hyperthyroidism and healthy adult controls, with selection following established inclusion and exclusion guidelines.

### 2.2 Inclusion criteria

1. Healthy Participants: Aged >18 years; Normal thyroid function.
2. Newly Diagnosed GD Patients: Aged >18 years; Meeting diagnostic criteria for GD hyperthyroidism; No prior antithyroid drug (ATD) therapy or treatment duration <1 month.
3. GD Patients Undergoing Treatment: Aged >18 years; with a ​​confirmed diagnosis of GD hyperthyroidism​​ and undergoing ​​consistent ATD therapy for at least 3 months​​. Based on treatment duration, patients will be categorized into four subgroups: ​​3, 6, 9, or 12 months of therapy​​.
4. GD Patients with Drug Withdrawal: Aged >18 years; Meeting diagnostic criteria for GD hyperthyroidism; Meeting GD-specific drug withdrawal criteria or self-discontinued medication (drug-free period >3 months).
5. GD Patients in Remission: Aged >18 years; Meeting diagnostic criteria for GD hyperthyroidism; Receiving regular ATD therapy (treatment duration ≥3 months); Achieving remission per GD treatment guidelines.
6. GD Patients with Relapse: Aged >18 years; Meeting diagnostic criteria for GD hyperthyroidism; Receiving regular ATD therapy (treatment duration ≥3 months); Meeting relapse criteria after standard therapy.

Diagnostic Criteria for GD Hyperthyroidism was established according to the Internal Medicine (9th Edition) criteria, encompassing the following: Mandatory Criteria: Clinical and biochemical evidence of hyperthyroidism: Documented history and clinical manifestations (hypermetabolic symptoms and signs).Biochemical confirmation via thyroid function tests (based on institutional laboratory standards: TSH <0.56 mIU/L, FT4 >16.02 pmol/L, and FT3 >7.37 pmol/L).Thyroid ultrasound findings: Diffuse thyroid lesions with increased vascularity, characterized by the “thyroid inferno” sign. Supportive Criteria: Positive TRAb (>1.75 IU/L) and/or TPOAb (>34 U/mL). Ophthalmic manifestations: Eyelid retraction, exophthalmos, or other infiltrative ophthalmopathy. Dermopathy: Pretibial myxedema or digital clubbing. A definitive diagnosis requires fulfillment of both mandatory criteria (1 and 2), with supportive criteria (3–5) providing additional confirmation.

Remission Criteria: Regular antithyroid drug (ATD) therapy for ≥6 months. Absence of thyrotoxicosis signs/symptoms for ≥3 months after ATD withdrawal. Normal thyroid function (FT3, FT4, and TSH within reference ranges). Decline in TRAb levels.

Drug Withdrawal Criteria: ATD therapy duration of 12–18 months (extended to 24–36 months for high-risk patients).Stable euthyroidism (TSH, FT3, and FT4 within reference ranges) at minimal effective ATD doses (e.g., methimazole 1–2.5 mg/day) for ≥3–6 months.

Relapse Criteria: Re-emergence of thyrotoxicosis symptoms in previously remitted GD patients. Biochemical hyperthyroidism (FT3 and FT4 above upper reference limits; TSH below lower reference limit).

Refractory GD Criteria: Persistent hyperthyroidism symptoms despite adequate ATD therapy (e.g., methimazole or propylthiouracil). Inability to taper ATD doses according to standard protocols. Recurrent relapses after achieving remission.

### 2.3 Exclusion criteria

1. Infants, children, women planning pregnancy within the next two years, pregnant or lactating women, and individuals with a history of multiple drug allergies. Hyperthyroidism secondary to nodular goiter or non-GD etiologies.
2. Grade III or higher thyroid enlargement, moderate-to-severe active thyroid-associated ophthalmopathy, thyrotoxic heart disease, or thyroid storm.
3. Moderate-to-severe hepatic dysfunction: alanine aminotransferase (ALT) or aspartate aminotransferase (AST) >3× upper limit of normal (ULN) or total bilirubin >34.2 μmol/L.
4. Moderate-to-severe renal impairment or end-stage renal disease: estimated glomerular filtration rate (eGFR) <60 mL/min/1.73m².
5. Severe treatment-related complications: peripheral white blood cell count <3.0×10^9^/L, neutrophils <1.5×10^9^/L, or severe drug hypersensitivity reactions.
6. Moderate-to-severe primary cardiovascular, cerebrovascular, digestive, or hematological disorders; autoimmune diseases (e.g., systemic lupus erythematosus, rheumatoid arthritis); inherited genetic disorders (e.g., chromosomal abnormalities, monogenic diseases); malignancies; or psychiatric disorders.
7. Recent exposure to iodinated contrast agents (e.g., coronary angiography, endoscopic retrograde cholangiopancreatography), iodine-containing medications (e.g., amiodarone), high-iodine diets (>50 g/day of iodine-rich foods such as seaweed or kelp), iodine-based antiseptics, or occupational iodine exposure.

The research protocol received ethical clearance from the Institutional Review Board at Harbin Medical University’s Second Hospital. Before study participation, all subjects provided signed consent forms. The investigation was conducted in full accordance with international ethical guidelines, particularly those established in the Helsinki Declaration, guaranteeing adherence to all required norms for human subject research and biological specimen handling.

## 3 Research methods

### 3.1 General information

Epidemiological questionnaires were used to collect basic information (name, gender, age, etc.), disease history (thyroid disease, other systemic diseases, etc.), iodine-rich food consumption, and allergy history.

### 3.2 Blood sample collection and preservation

10mL venous blood was collected using a disposable vacuum procoagulant tube. After 30 minutes at room temperature, the serum was separated by centrifugation at 3000r/rpm for 10 minutes, and the upper serum was transferred to Eppendorf Polypropylene (EP) tubes. After labeling and classification, it was sealed and stored in the refrigerator at -80°C.

### 3.3 Thyroid function parameter assays

The serum TSH, FT3, FT4, TT3, TT4, TPOAb, and TgAb levels were determined by electrochemiluminescence immunoassay. The reagents were the original kits of the instrument. The detection instrument and reference value range in our center are shown in **Table 1**.

**Table 1:** Reference ranges for thyroid hormones and antibody markers.

Parameters	Thyroid-related indices	Reference ranges	Analytical instruments
Thyroid Hormones	FT3	3.53-7.37(pmol/L)	DXI800
	FT4	7.98-16.02(pmol/L)	DXI800
	TSH	0.56-5.91(uIU/mL)	DXI800
	TT3	0.92-2.38(nmol/L)	DXI800
	TT4	69.71-163.95(nmol/L)	DXI800
Thyroid Antibodies	TgAb	0-4(IU/mL)	DXI800
	TPOAb	0~9(IU/mL)	DXI800
	TRAb	0-1.5(IU/L)	MAGLUMI X8

### 3.4 Thyroid volume measurement

A SAMSUNG RS80A-6 three-dimensional ultrasound instrument with a probe frequency of 7.5MHZ was used to sweep up and down the thyroid in the order of left lobe, right lobe, and isthmus. The volume of the unilateral thyroid lobe was calculated by the formula: V=0.479×W×D×L/1000, Tvol was the sum of the volume of left and right lobes, and the volume of thyroid isthmus was ignored. If it exceeds the normal reference standard of WHO or China, it is judged as goiter (“ Diagnostic Criteria of endemic goiter “(WS 276-2007) (normal value of Tvol: adult (female) ≤18.0mL, adult (male) ≤25.0mL)).

### 3.5 Serum total iodine measurement

**Figure 2:**
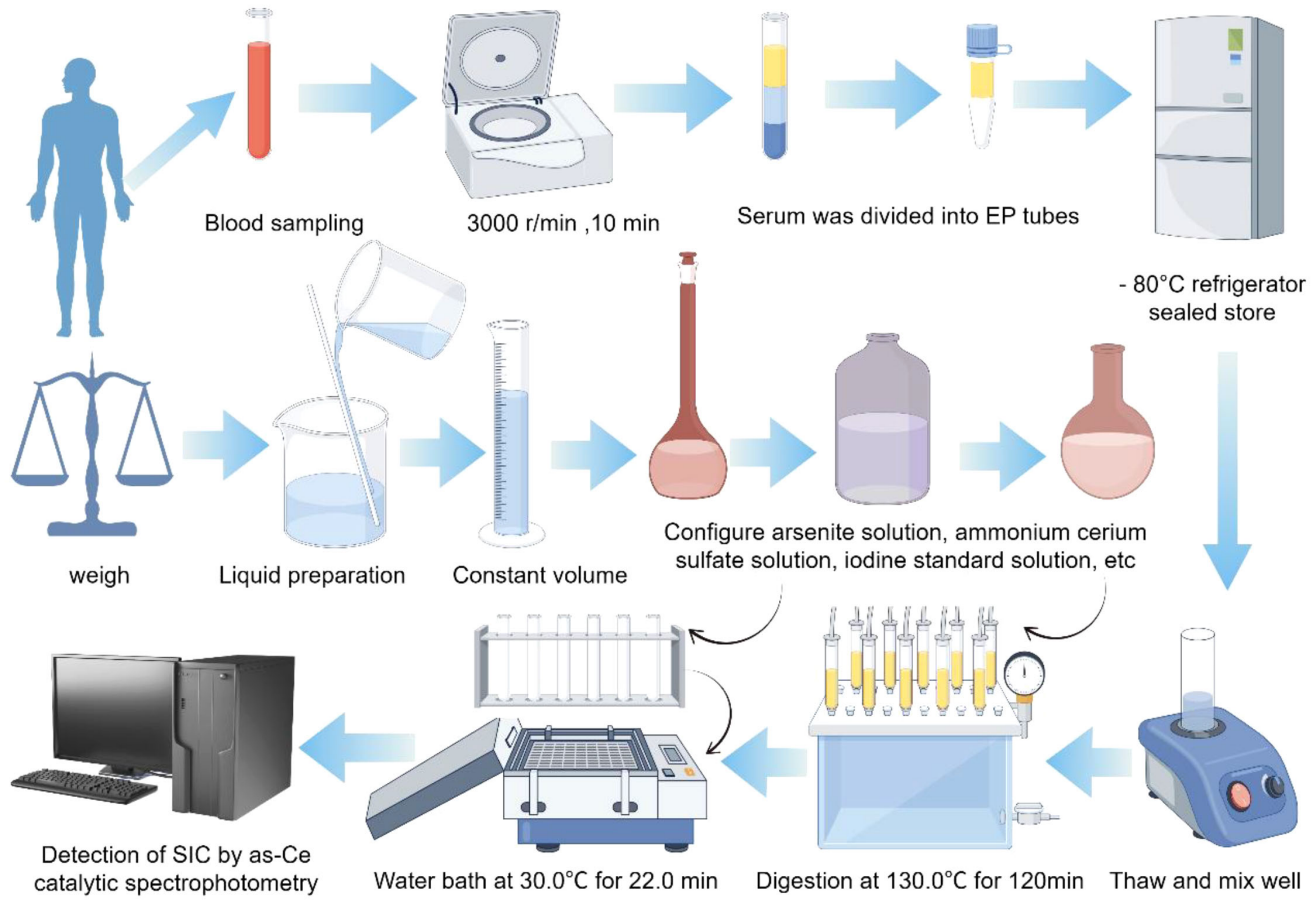
Experimental procedure flowchart.

In this study, the determination of total iodine concentration in serum was carried out by using the **“**Arsenic-cerium Catalytic Spectrophotometric Method**”** (WS/T 572-2017), which complies with the national health industry standard. According to the WHO (9, 10) the recommended by SIC reference range is divided into class high blood iodine (> 90 mu g/L), optimal blood iodine (45–90 mu g/L), and low blood iodine (< 45 mu g/L) three categories (**Figure 2**; **Table 2**).

**Table 2:** Reaction time of the arsenic-cerium catalytic reaction at different temperatures.

Temperature/°C	Reaction time/min	Temperature/°C	Reaction time/min
15	61	23	36
16	57	24	33
17	54	25	31
18	50	26	29
19	47	27	27
20	43	28	26
21	41	29	24
22	38	30	22

### 3.6 Sample quality control

**Figure 3:**
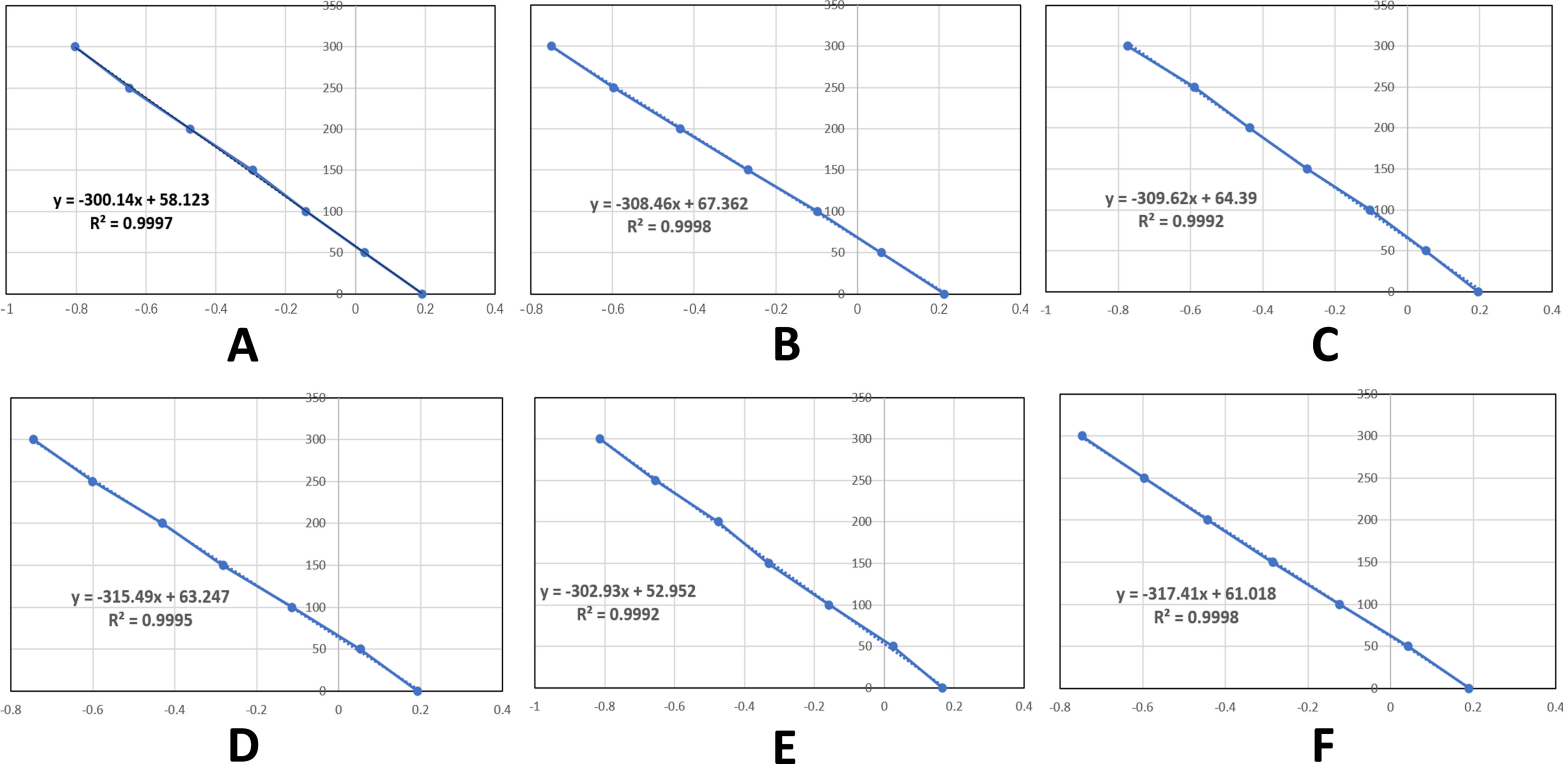
Standard curves for each assay batch. **(A-F)** respectively represent the standard curves of serum iodine detection from batch 1 to Batch 6.

​​Pre-Analytical Procedures: Serum samples were collected, transported, and stored in ​​iodine-free containers​​ to minimize ​​exogenous iodine contamination​​.Analytical Quality Control: Each assay batch incorporates Certified reference materials (CRM, National Institute of Metrology, China)​​ to ensure ​​measurement accuracy​​.Freshly calibrated standard curves​​, with a ​​correlation coefficient (R²≥0.999​​ (representative curves illustrated in ​​**Figure 3**​​).

## 4 Data processing

### 4.1 Data management and analysis

Data collection and validation were performed using Microsoft Excel 2019 (Microsoft Corporation, USA). Statistical processing was conducted with IBM SPSS Statistics (Version 22.0, IBM Corp.). Flowcharts were created using FigDraw (Version 2.0, FigDraw Research Group). Graphical representations were generated through the CNSKnowall scientific visualization system (Version 2.3.1, CNSKnowall Inc.).

### 4.2 Statistical analysis

Continuous variables were assessed for normality using the Kolmogorov-Smirnov test. Normally distributed data (e.g., age, FT4) were reported as mean ± SD and compared using t-tests (two groups) or ANOVA (multiple groups). Non-normal data (e.g., TRAb) were expressed as median (IQR) and analyzed with Mann-Whitney U or Kruskal-Wallis tests. Categorical data were presented as n (%) and compared using *χ²* or Fisher’s exact tests. The SIC reference range was established using the 2.5th-97.5th percentile method. Correlations were evaluated using Pearson’s (normal data) or Spearman’s (non-normal data) tests to examine SIC-thyroid function relationships. Statistical significance was set at p<0.05 (two-tailed).

## 5 Results

### 5.1 Comparison of baseline demographic and clinical characteristics

A total of 430 GD patients and 80 healthy adults were enrolled in this study. The GD patients were predominantly young and middle-aged females, with females accounting for 75.12% (323/430) of the 430 GD patients, and healthy adults (n=80) were older than untreated GD patients (n=80) (40.46 ± 12.52 vs. 34.18 ± 11.65 years, P<0.05) (**Table 3**).

### 5.2 Serum iodine concentration profiles

The study analyzed 510 serum samples, including 80 serum samples from normal adult euthyroid individuals and 430 serum samples from adult Graves’ disease (GD) patients. Among the GD patients, 80 were newly diagnosed GD patients who had not been treated, with an SIC value of 100.15 ± 40.25 μg/L, ranging from 27.76 to 187.2 μg/L, and a 95% CI of 36.49 to 165.67 μg/L; another 350 GD patients were receiving antithyroid drug treatment, with an SIC value of 65.74 ± 19.37 μg/L, ranging from 28.33 to 114.97 μg/L, and a 95% CI of 32.84 to 92.02 μg/L. The SIC in untreated GD patients was significantly higher than that in treated GD patients (Mann-Whitney U test, P < 0.05). However, no significant difference was found between healthy adults and treated GD patients (P = 0.557).

The study tracked the serum iodine concentration (SIC) of patients with Graves’ disease (GD) at different treatment stages: After 3 months of treatment, the mean SIC reached 58.63 μg/L (range: 28.33-114.97), at 6 months the SIC rose to 65.03 μg/L (range: 30.09-135.28), at 9 months the SIC slightly increased to 67.04 μg/L (range: 32.79-124.60), and at 12 months the SIC reached 75.28 μg/L (range: 33.25-108.52). During the treatment transition period: The average SIC during the discontinuation phase was 69.04 μg/L (range: 24.21-139.02), the SIC decreased to 62.74 μg/L (range: 26.02-107.78) during the remission period, and the SIC increased to 79.26 μg/L (range: 31.24-142.72) during the recurrence period. The main conclusions drawn from the statistical analysis were: The SIC of GD patients without treatment was significantly higher than that of all treated groups (P<0.05), there was no significant difference between healthy adults and patients in the early treatment or remission period (P>0.05), and the SIC at the 12th month and during the discontinuation period was higher than that in previous treatment periods (P<0.05) (**Table 3**).

**Table 3:** SIC and medical reference ranges across study populations.

Grouping	Female,n (%)	Age (years)	SIC (μg/L)	SIC (min,max)	95%CI (P2.5,P97.5)
Normal (n=80)	54	40.46 ± 12.52	63.78 ± 18.92^de^	(28.15, 129.74)	(31.40, 95.54)
GD(untreated) (n=80)	63	34.18 ± 11.65	100.15 ± 40.25^a^	(27.76, 187.2)	(36.49, 165.67)
GD(3 months) (n=50)	37	39.02 ± 1.61	58.63 ± 17.57^e^	(28.33, 114.97)	(32.84, 92.02)
GD(6 months) (n=50)	38	36.06 ± 11.81	65.03 ± 21.88^de^	(30.09, 135.28)	(38.96, 126.33)
GD(9 months) (n=50)	38	38.74 ± 11.51	67.04 ± 20.69^cde^	(32.79, 124.60)	(39.48, 112.19)
GD(12 months) (n=50)	34	36.08 ± 12.66	75.28 ± 15.74^bc^	(33.25, 108.52)	(40.84, 104.99)
Withdrawal period (n=50)	37	40.28 ± 14.54	69.04 ± 25.88^cd^	(24.21, 139.02)	(40.84, 104.99)
Remission period (n=50)	40	42.66 ± 12.34	62.74 ± 2.39^de^	(26.02, 107.78)	(35.05, 92.32)
Relapse period	36	36.38 ± 10.12	79.26 ± 24.86^b^	(31.24, 142.72)	(41.05, 136.77)

Different superscript letters denote statistically significant differences in SIC between groups. Groups sharing at least one common letter (e.g., “a” and “ab”) indicate no significant difference. For example: “a” vs. “ab”: Share the letter “a” → no difference (P ≥ 0.05).” ab” vs. “bc”: Share the letter “b” → no difference.” a” vs. “bc”: No shared letters → significant difference (P < 0.05).

### 5.3 Correlation between SIC and thyroid function indices

In 80 normal thyroid adults, SIC was positively correlated with FT4 (r=0.311, P<0.001), but had no significant correlation with FT3, TPOAb, or TgAb (P>0.05; **Table 4**). In newly diagnosed, untreated GD patients (n=80), SIC was positively correlated with FT4 (r=0.213, P=0.024) and TRAb (r=0.369, P<0.001), but had no correlation with FT3, TPOAb, or TgAb (P>0.05). SIC was positively correlated with left lobe volume (r=0.41), right lobe volume (r=0.38), and total thyroid volume (r=0.43) (P<0.001; **Table 5**).

**Table 4:** Correlation analysis between SIC and thyroid function indices in euthyroid adults (n = 80).

Thyroid Function indices	Measured values	Correlation coefficients (r)	P Values
FT3 (pmol/L, $\bar{x}\pm s$ )	4.47 ± 1.49	0.121	0.379
FT4 (pmol/L, $\bar{x}\pm s$ )	10.63 ± 5.63	0.311	**<0.001**
TSH (mU/L, $\bar{x}\pm s$ )	2.42 ± 0.98	0.961	0.397
TgAb [IU/ml,M(Q1,Q3)]	2.40(1.93,21.03)	-0.006	0.955
TPOAb[IU/ml,M(Q1,Q3)]	3.48(0.96,6.30)	0.064	0.574
TRAb(IU/L, $\bar{x}\pm s$ )	0.076 ± 0.038	0.162	0.151

M, Median; Q1/Q3, First/Third Quartile, Correlation Coefficient (r): Very high correlation: 0.9<r<1.0. Strong correlation: 0.7≤r<0.9, Moderate correlation: 0.4≤r<0.7. Weak correlation: 0.2≤r<0.4. Negligible/no correlation: 0.0≤r<0.2. The bolded P value indicates statistical significance.

**Table 5:** Correlation analysis between SIC and thyroid function indices in newly diagnosed untreated GD patients (n = 80).

Thyroid Function Indices	Measured values	Correlation coefficients (r)	P Values
FT3 (pmol/L, $\bar{x}\pm s$ )	25.62 ± 10.05	-0.060	0.597
FT4 (pmol/L, $\bar{x}\pm s$ )	56.79 ± 21.17	0.213	**0.024**
TSH (mU/L, $\bar{x}\pm s$ )	0.009 (0.003,0.010)	-0.185	0.100
TgAb [IU/ml,M (Q1,Q3)]	68.38 (2.95,221.90)	0.320	0.143
TPOAb [IU/ml,M (Q1,Q3)]	118.50 (10.88,683.43)	0.151	0.184
TRAb (IU/L, $\bar{x}\pm s$ )	23.59 ± 12.73	0.369	**<0.001**
TT3 (nmol/L, $\bar{x}\pm s$ )	6.92 ± 2.43	-0.175	0.121
TT4 (nmol/L, $\bar{x}\pm s$ )	313.84 ± 41.06	0.118	0.296
Left lobe (mm)	10.59 ± 4.75	0.360	**0.001**
Ringht lobe (mm)	5.29 ± 0.81	0.333	**0.003**
Tvol (mL)	21.89 ± 9.09	0.412	**0.000**
Isthmus (mm)	0.43 ± 0.16	0.017	0.882

M, Median; Q1/Q3, First/Third Quartile, Correlation Coefficient (r): Very high correlation: 0.9<r<1.0. Strong correlation: 0.7≤r<0.9, Moderate correlation: 0.4≤r<0.7. Weak correlation: 0.2≤r<0.4. Negligible/no correlation: 0.0≤r<0.2. The bolded P value indicates statistical significance.

**Figure 4:**
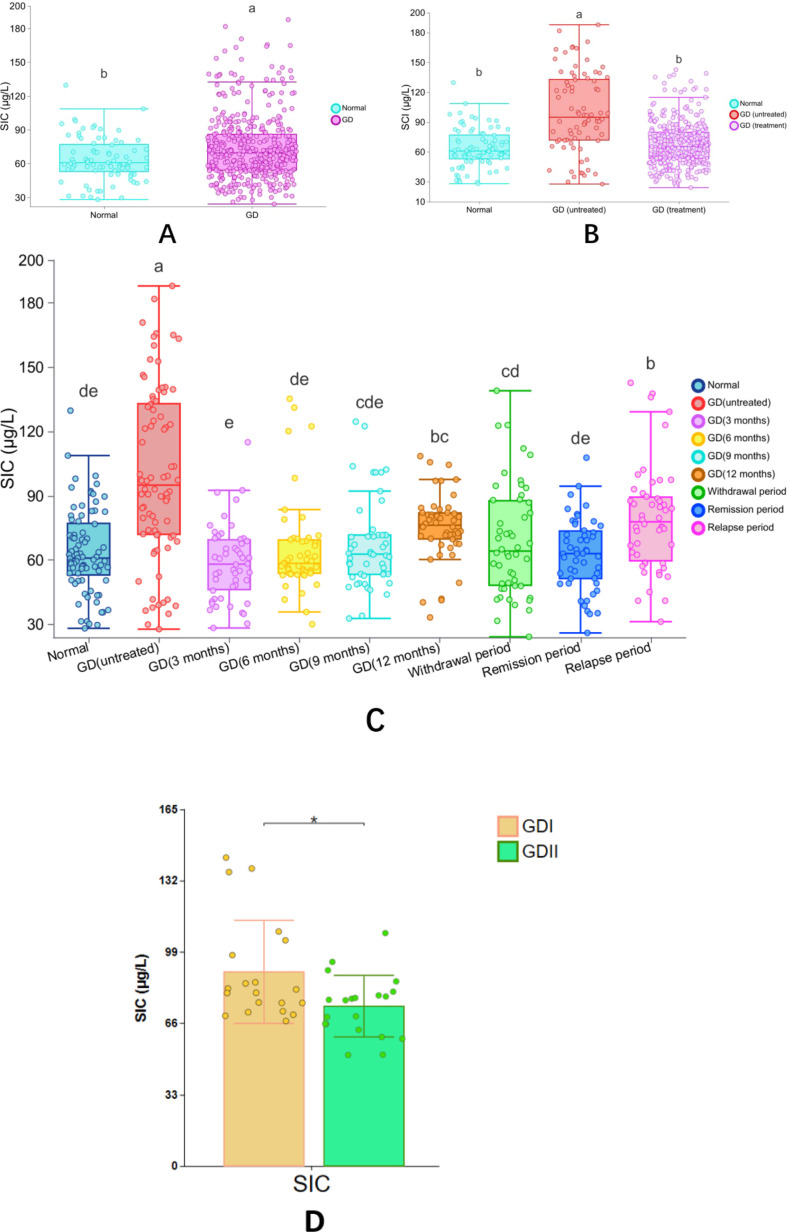
Correlation heatmap of SIC and thyroid function indices in GD patients across treatment phases. panel descriptions: **(A–D)** Correlation heatmaps for GD patients at 3-, 6-, 9-, and 12-month therapy phases, respectively. **(E)** Correlation heatmap during the remission phase. **(F)** Correlation heatmap during the relapse phase. Heatmap Features: Color-coded visualization of pairwise correlations (red: positive; blue: negative) across multiple variables (e.g., SIC, FT4, TRAb). In-built statistical significance markers: *P < 0.05. **P < 0.01. ***P < 0.001. Correlation strength scales with color intensity (see legend).

The correlation of GD patients in different stages of treatment: 3-month treatment, SIC was not significantly correlated with thyroid function index (P>0.05). 6-month treatment: FT4 was positively correlated (r=0.27, P=0.013), and the other indicators were not correlated. 9-month treatment: FT4 was positively correlated (r=0.31, P=0.008), TRAb was positively correlated (r=0.25, P=0.026). 12-month treatment: FT3 was positively correlated (r=0.29, P=0.011), FT4 was positively correlated (r=0.33, P=0.004). Remission period: FT4 was positively correlated (r=0.22, P=0.047). Relapse period: FT4 was positively correlated (r=0.36, P=0.002), and TRAb was positively correlated (r=0.33, P=0.006). Other indicators (FT3, TPOAb, TgAb) were not significantly correlated in all stages (P>0.05) (**Figure 4**).

### 5.4 Distribution of SIC categories by percentage

**Figure 5:**
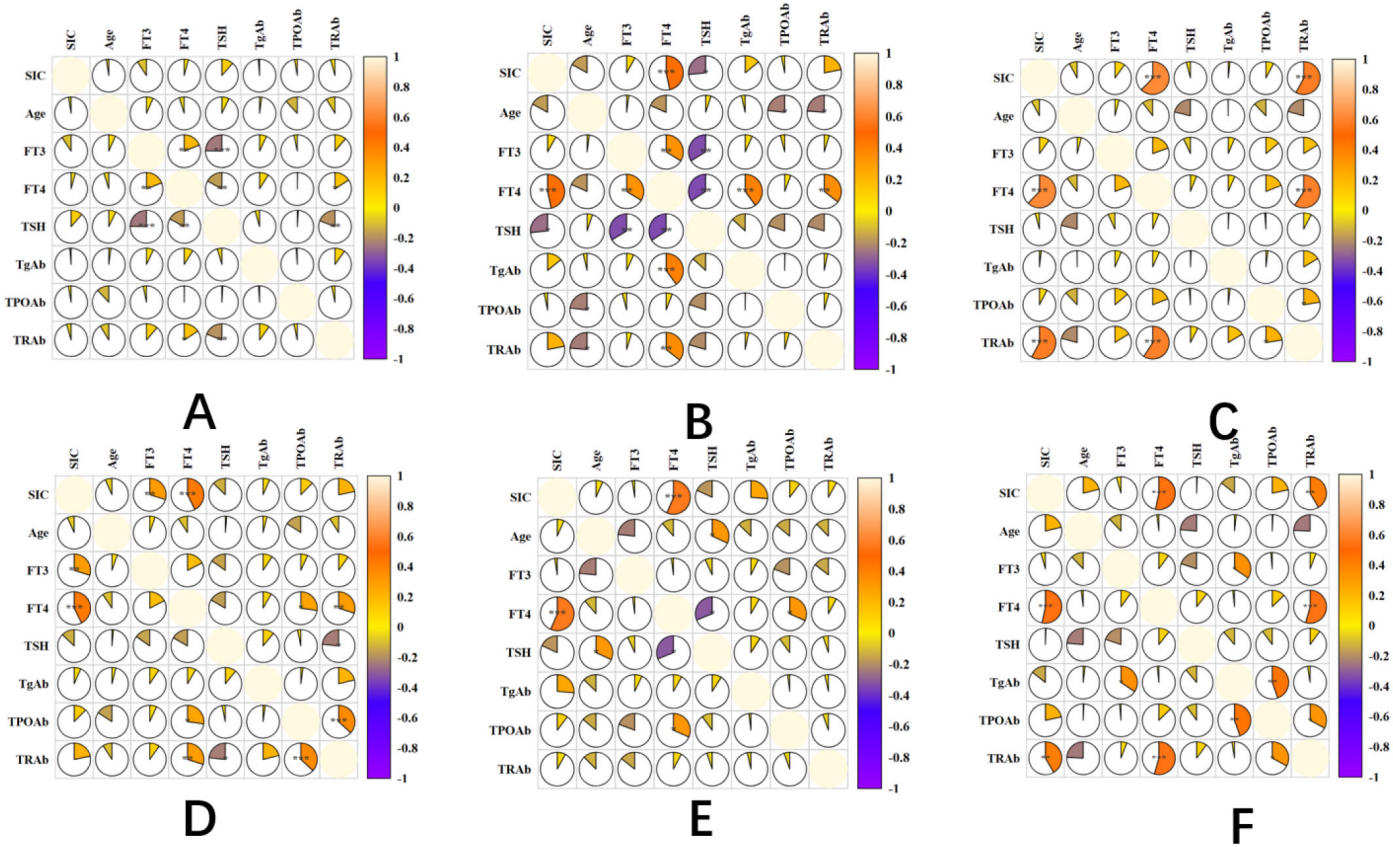
Distribution of SIC across population groups based on WHO-defined reference ranges thyroid antibody positivity rate in untreated newly diagnosed GD patients.

According to the SIC reference range specified by the WHO, the subjects were divided into three groups: iodine deficiency (<45μg/L), appropriate iodine (45-90μg/L), and excessive iodine (>90μg/L). Compared with healthy adults, the incidence of excessive iodine (>90μg/L) in newly diagnosed GD patients who had not been treated was significantly higher (61.25% vs 11.25%, P<0.05), while the incidence of appropriate iodine (45-90μg/L) was significantly lower (27.50% vs 76.25%, P<0.05). There was no significant difference in the incidence of iodine deficiency (<45μg/L) (11.25% vs 12.50%, P>0.05). There was no statistically significant difference in the distribution of SIC at different treatment stages of GD patients (P>0.05). See **Table 6** and **Figure 5** for details.

**Table 6:** SIC category distribution across study cohorts.

Population Grouping	Number of Cases	SIC ( $\bar{x}$ ± SD,μg/L)	SIC Category Distribution (n,%)		
			<45μg/L	45-90μg/L	>90μg/L
Normal	80	63.78 ± 18.9^de^	(10/80) 12.50%	(61/80) 76.25%	(9/80) 11.25%
GD (untreated)	80	100.15 ± 40.25^a^	(9/80) 11.25%	(22/80) 27.50%	(49/80) 61.25%
GD (3 months)	50	58.63 ± 17.57^e^	(11/50) 22.00%	(35/50) 70.00%	(4/50) 8.00%
GD (6 months)	50	65.03 ± 21.88^de^	(8/50) 16.00%	(36/50) 72.00%	(6/50) 12.00%
GD (9 months)	50	67.04 ± 20.69^cde^	(3/50) 6.00%	(39/50) 78.00%	(8/50) 16.00%
GD (12 months)	50	75.28 ± 15.74^bc^	(5/50) 10.00%	(38/50) 76.00%	(7/50) 14.00%
Withdrawal period	50	69.04 ± 25.88^cd^	(11/50) 22.00%	(33/50) 66.00%	(11/50) 22.00%
Remission period	50	62.74 ± 2.39^de^	(10/50) 19.00%	(37/50) 74.00%	(3/50) 7.00%
Relapse period	50	79.26 ± 24.86^b^	(6/50) 12.00%	(30/50) 60.00%	(14/50) 28.00%

Categorical data were presented as n (%) and compared using *χ²* or Fisher’s exact tests.

**Figure 6:**
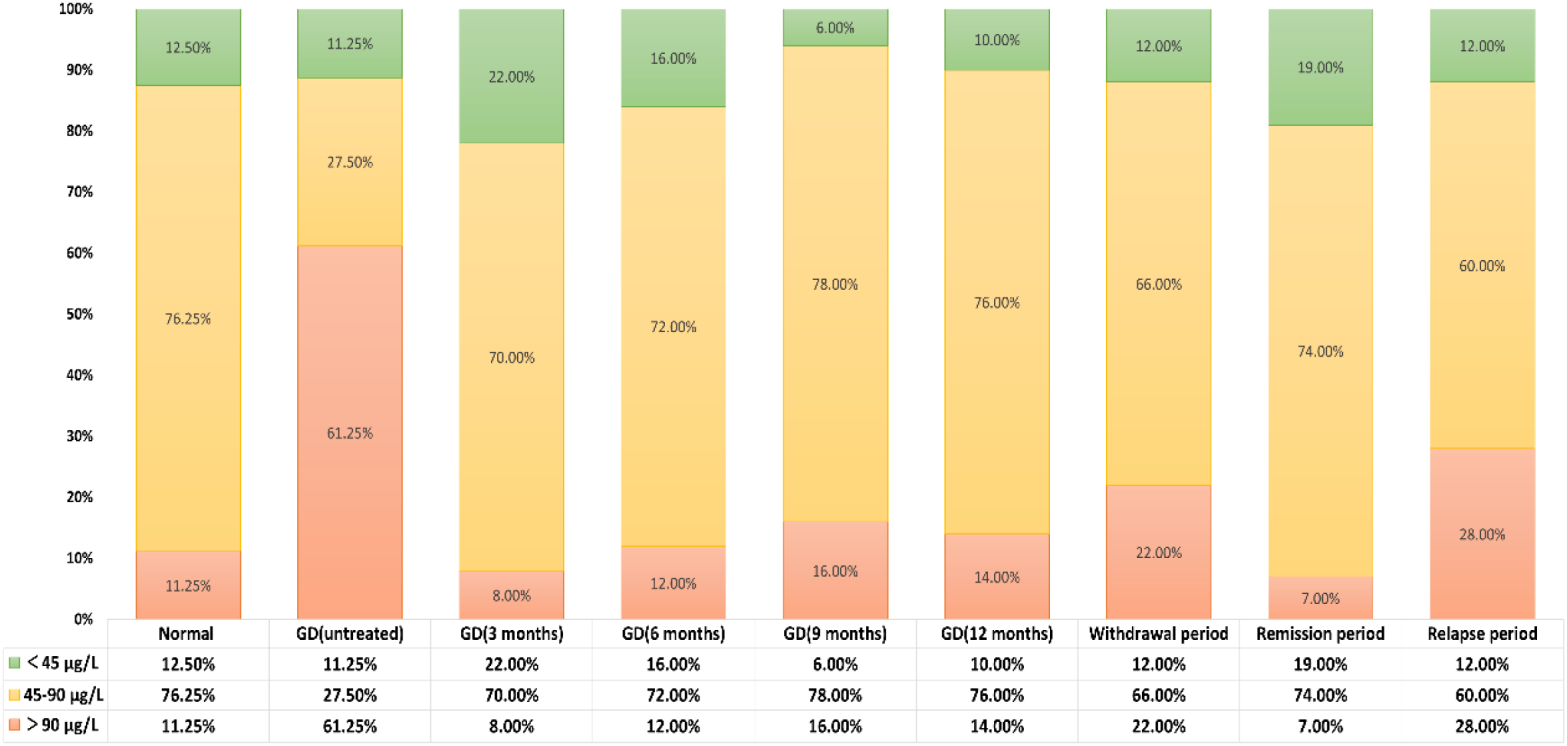
SIC in hyperthyroid GD patients across treatment phases and euthyroid adults. **(A)** Comparison between euthyroid adults and GD patients (background cohort). **(B)** Comparison among euthyroid adults, untreated GD patients, and GD patients under therapy. **(C)** Comparison between euthyroid adults and GD patients across distinct treatment phases. **(D)** Comparison between refractory GD and standard-therapy-responsive GD patients. Statistical Analysis: *Post hoc* comparisons were performed using Fisher’s least significant difference (LSD) test (selected from five algorithms provided by CNSKnowall: Fisher’s LSD, Tukey’s HSD, Duncan, Scheffé, and Games-Howell). Rationale for LSD selection: Suitable for small sample sizes, homogeneity of variance (Levene’s test P > 0.05), and normally distributed residuals (Shapiro-Wilk P > 0.05). Significance Labeling: Superscript letters: Groups sharing at least one common letter (e.g., “a” and “ab”) indicate no significant difference (P ≥ 0.05). Example: “a” vs. “ab”: Shared letter “a” → no difference.” ab” vs. “bc”: Shared letter “b” → no difference.” a” vs. “bc”: No shared letters → significant difference (P < 0.05). Asterisk (*): Denotes P < 0.05 for pairwise comparisons.

There is a difference in the positivity rate of TPOAb and TGAb in newly diagnosed GD patients with hyperthyroidism, but this difference is not significant (*χ²*=1.129, P=0.569). The study shows that a lower SIC level increases the positivity rate of TgAb and TPOAb, with TPOAb levels significantly higher in the low iodine group compared to the high iodine and appropriate iodine groups (P<0.05). Moreover, the positivity rate of TPOAb in the low iodine group is much higher than that in the high iodine and appropriate iodine groups (P<0.05). The concentration of TGAb is greater in the high iodine group than in the low iodine and appropriate iodine groups (P<0.05), but there is no significant difference in the positivity rate of TGAb between the low iodine and high iodine groups, as well as the appropriate iodine group (P>0.05) (**Figure 6**; **Table 7**).

**Table 7:** Thyroid antibody positivity rate in untreated newly diagnosed GD patients.

WHO Classification	TPOAb		TGAb	
	Concentration (IU/ml)	Positivity Rate (%)	Concentration (IU/ml)	Positivity Rate (%)
High iodine group (n=61)	95.29 (7.33,587.38)	(19/49) 38.78%	69.52 (3.52,194.44)	(11/49) 22.45%
Adequate iodine group (n=10)	50.40 (5.44,417.16)	(8/22) 36.36%	18.09 (2.55,137.86)	(6/22) 27.27%
Low iodine group (n=9)	406.94 (406.94,855.4)	(6/9) 66.67%	25.73 (0.77,195.78)	(4/9) 44.45%

Categorical data were presented as n (%) and compared using *χ²* or Fisher’s exact tests.

## 6 Discussion

Graves’ disease (GD) is a systemic autoimmune disease characterized by the presence of pathogenic antibodies (TRAbs) against the thyroid-stimulating hormone (TSH) receptor, which activate the TSH receptor on the surface of thyroid cells, leading to excessive secretion of thyroid hormones (T3, T4) and the onset of hyperthyroid symptoms. Iodine, as a key raw material for the synthesis of thyroid hormones, must be maintained within an appropriate range. Excessive intake of iodine beyond physiological needs (urinary iodine > 300 μg/day) can cause abnormal post-translational modification of thyroid globulin (Tg), exposing epitopes previously hidden and breaking down the body’s original immune tolerance state. It should be emphasized that a significant increase in iodine intake, i.e., urinary iodine > 500 μg/day, may trigger GD or iodine-induced hyperthyroidism (Jod-Basedow phenomenon) (11). The interaction between hyperthyroidism associated with GD and iodine nutritional status has become a key issue in endocrine research.

Iodine deficiency disorders (IDDs), such as endemic goiter, have become relatively rare in China since the implementation of the national iodized salt policy. People’s overall iodine nutritional status has also reached the World Health Organization’s recommended standard, that is, the urine iodine concentration (UIC) is between 100-199 μg/L. Some studies have shown that there may be an interaction between iodine and autoimmune thyroid diseases. Some patients have experienced worsening or recurrence of their condition after increasing their iodine intake, and a higher incidence of GD-related hyperthyroidism has been found in areas with sufficient iodine (12). Furthermore, new research indicates that during the treatment of GD with antithyroid drugs, appropriate iodine intake is better than complete iodine restriction, as this can reduce the recurrence rate, while overly strict dietary iodine control is not beneficial (13). Therefore, dynamic monitoring of iodine nutritional status and precision management of iodine intake are critical for GD patients with hyperthyroidism, avoiding a “one-size-fits-all” approach that risks excessive iodine exposure in certain subpopulations.

SIC is an important intermediate product reflecting iodine absorption and metabolism, playing a key role in thyroid hormone synthesis and function. Relevant research shows that it is of great significance for the diagnosis of thyroid diseases because of its high stability, which can truly reflect the long-term iodine nutritional status. Therefore, this study mainly explores the iodine nutritional status and its relationship with thyroid function indicators of GD-related hyperthyroid patients at different treatment stages and healthy adults. In this northern city of Harbin, the arsenic cerium catalytic photometric method specified in the National Health and Health Commission of China’s standard (WS/T 107.1-2016) was adopted to implement the investigation of SIC content. Although Harbin belongs to the iodine-sufficient area of our country, there are still significant individual differences in the expression of iodine status (deficiency, normal, or excessive). Especially for untreated GD hyperthyroid patients, their SIC levels are significantly higher than those in the healthy control group (P<0.05). Previous studies have reported a U-shaped relationship between iodine intake and GD, with Mostbeck et al. reporting that the proportion of hyperthyroidism increased by 12% after the addition of salt iodization (14), which is consistent with our results. Moreover, we found that most patients are located on the right side of the U-shaped curve (i.e., the high iodine side), suggesting that the onset of GD may be more related to iodine overexposure. However, the specific biological mechanism of SIC and thyroid dysfunction is still unclear.

Regional studies investigate the SIC and its association with thyroid function indicators. A study in Guangxi involving 465 adults with normal thyroid function showed that their average UIC value reached 191.0 μg/L (indicating that the area belongs to the category of iodine sufficiency), while the SIC was 64.26 ± 22.20 μg/L, and a reference range for clinical use was provided with a high probability, ranging from 26.33 to 116.35 μg/L (15). In Liaoning Province, among 1621 adults with normal thyroid function, the SIC level was 62.0μg/L (IQR: 53.5-71.0), with a 95% reference range of 37.0-103.0μg/L (16). In the Beijing urban area, the SIC levels of iodized salt consumers ranged from 39 to 179μg/L, and no association was found between SIC and UIC, T3, T4, or TSH levels (17). In a study of 149 adults with normal thyroid function in Fujian Province, the median UIC was 119.45μg/L (iodine adequate range: 100-199μg/L), and the SIC was 61.35 ± 16.20μg/L (95% reference range: 34.36-97.17μg/L). SIC was weakly positively correlated with FT4 (18). In Heilongjiang Province, among 224 adults with normal thyroid function, the SIC level was 66.32 ± 15.46μg/L (95% reference range: 36.02-96.62μg/L), and SIC was positively correlated with FT4, but not with FT3 or TSH (19). This study further analyzed 80 healthy adults in Heilongjiang Province, with a SIC level of 63.78 ± 18.92μg/L (95% CI: 31.40-95.54μg/L), which met the WHO-defined iodine sufficient range. SIC and FT4 show a significant positive correlation, while there is no correlation with FT3, TPOAb, and TgAb, which is consistent with previous reports. The increase in FT4 is one of the important characteristics of hyperthyroidism. When serum iodine content increases, thyroid follicular cells utilize an “iodine pump” to efficiently absorb iodine and synthesize more FT4 (20). The positive correlation between serum iodine and FT4 can indirectly reflect the iodine nutritional status of the population. Therefore, the change in FT4 after adjusting iodine intake can provide a reference for personalized iodine supplementation for patients with hyperthyroidism. TRAb is a marker antibody for Graves’ disease, with a positive rate of 80%-95%. The positive correlation between serum iodine levels and TRAb may indicate the impact of iodine intake on AITD (21). A high-iodine environment may enhance the iodination degree of thyroglobulin and promote the production of TRAb, thereby increasing the risk of GD. This study further identified positive correlations between SIC and the left thyroid lobe volume (r=0.360), right thyroid lobe volume (r=0.333), and total thyroid volume (Tvol) (r=0.412), with all P-values <0.001. However, Tvol exhibited limited sensitivity to short-term fluctuations in iodine status, demonstrating significant changes only under prolonged iodine deficiency or excess. Thus, Tvol primarily reflects long-term iodine nutritional status.

This study revealed that the median SIC in adults with GD was 98.05 μg/L (IQR: 84.68–113.19 μg/L), slightly higher than previously reported values for iodine-sufficient pregnant women (86.74 μg/L, IQR: 74.99–100.15 μg/L) and healthy adults (66.32 ± 15.46 μg/L) in urban Harbin (22). Among 430 GD hyperthyroidism patients analyzed using the standardized arsenic-cerium catalytic spectrophotometric method, SIC levels were significantly elevated compared to healthy controls (*P*<0.05). A cross-sectional study covering six provinces in China showed that the risk of overt hyperthyroidism in adults with SIC > 97.59 μg/L increased by 21 times (23). Considering the overall iodine sufficiency status in Harbin (median urinary iodine: 100-199 μg/L), the presence of high SIC in GD patients may reflect disease-specific or treatment-related iodine metabolism abnormalities, indicating a direct link between high iodine exposure and thyroid dysfunction. A longitudinal study showed that from 2009 to 2015, the median urinary iodine decreased from 219.7 μg/L to 175.9 μg/L, and the incidence of overt hyperthyroidism, subclinical hyperthyroidism, GD, and goiter all decreased (P < 0.05) (24), emphasizing the importance of monitoring SIC and precise iodine management for newly diagnosed GD patients.

We further investigated SIC dynamics across GD treatment phases. Untreated *de novo* GD patients had an SIC of 100.15 ± 40.25 μg/L (range: 27.76–187.2 μg/L; 95% CI: 36.49–165.67 μg/L). During regular treatment at 3, 6, and 9 months, SIC decreased significantly to iodine-sufficient levels, likely attributable to antithyroid drug adherence and dietary iodine restriction. However, at 12 months of treatment and during relapse, SIC rebounded significantly compared to other phases (*P*<0.05), though remaining within the adequate range. This may reflect relaxed dietary iodine control or relapse-driven metabolic alterations. These findings highlight the necessity of concurrent monitoring of thyroid function and iodine status during maintenance therapy to identify contributing factors and optimize treatment strategies.

Research shows that excessive iodine intake increases antibody titers, autoreactive T cells, and dendritic cell numbers, thereby exacerbating autoimmune reactions in susceptible populations (25). There is an association between excessive iodine intake and an increased rate of thyroid autoantibody positivity, which may be caused by oxidative stress-induced damage to thyroid cells. This prompts the immune system to activate and release autoantigen substances such as thyroid peroxidase or thyroid-stimulating hormone receptors. This process produces free radicals, which can exert cytotoxic effects on thyroid cells or directly stimulate lymphocytes, leading to an increased frequency and recurrence of GD (26). Moreover, reports are indicating an interaction between excessive iodine intake and vitamin D deficiency, which can affect the positive expression of TRAb in early pregnancy (25). Women with positive TRAb have higher total thyroxine levels (27). In this study, we observed a significant positive correlation between SIC and TRAb titers in GD patients (r=0.369, *P* < 0.001). This finding may be attributed to excessive iodine intake triggering aberrant activation of TRAb, which sustains T- and B-lymphocyte activity, disrupts immune homeostasis, and ultimately exacerbates GD-associated hyperthyroidism.

Studies have also shown that when SIC is suboptimal (either low or high), the positivity rates of TPOAb and TgAb increase progressively with elevated iodine intake. High iodine levels may promote dendritic cell maturation, enhance the antigenicity of Tg, and elevate TgAb titers by transforming thyroid epithelial cells into antigen-presenting cells (28). Animal experiments also indicate that iodine can cause thyroid autoimmunity. Excessive iodine can lead to spontaneous thyroiditis in genetically susceptible animals. The possible mechanism is that T cells recognize Tg. In untreated newly diagnosed GD patients, the TgAb level in the high iodine group is higher than that in the low iodine and appropriate iodine groups. However, the positive rates of TgAb and TPOAb in the low iodine group are both higher than those in the high iodine group. This study also shows that the TPOAb concentration and positive rate in the low iodine group are both higher than those in the high iodine and appropriate iodine groups (P<0.05). The possible mechanism for iodine deficiency leading to an increased positive rate of TPOAb: In the state of iodine deficiency, the iodination process of Tg is blocked, causing the accumulation of intermediate products in the synthesis of thyroid hormones and the disorder of the synthetic pathway. Simultaneously, the REDOX balance within thyroid cells is disrupted, ROS accumulates, and the antioxidant defense system is damaged, thereby triggering or exacerbating autoimmune reactions against thyroid antigens (29, 30). Moreover, under iodine deficiency conditions, the molecular conformation and epitope accessibility of TPO undergo significant changes, with previously hidden TPO epitopes exposed. These newly emerged epitopes can be recognized by B cells and trigger the generation of TPOAb (31). Clinical studies have found that in populations with long-term iodine deficiency, antibodies against multiple TPO epitopes can be detected, which not only cause epitope expansion and cross-immune responses but may also lead to changes in clinical phenotypes, such as the transition from Graves’ disease to Hashimoto’s thyroiditis (12, 32).

This study establishes and describes the medical reference intervals for adult SIC under different thyroid conditions, providing a scientific basis for personalized iodine nutrition evaluation. For patients with GD hyperthyroidism, precise iodine management can be achieved by using SIC to evaluate iodine during the treatment period: during the active phase, patients with higher SIC values need to strictly control their iodine intake (such as using non-iodized salt and avoiding high-iodine foods like kelp and seaweed); during the remission phase, it is important to maintain iodine intake within an appropriate range, regularly check SIC, TRAb, and thyroid function, thereby better planning iodine supply throughout life and preventing excessive iodine supplementation or over-restriction of iodine (33).

Research limitations: Regional specificity: The study was conducted in areas with sufficient iodine, with the vast majority of participants having sufficient or even excessive iodine intake; single-center recruitment: Recruitment was only conducted at the Second Affiliated Hospital of Harbin Medical University, making it difficult to be universally applicable; cross-sectional design: There is a lack of follow-up or repeated observations on the same group of people, and future studies can conduct prospective studies to confirm the dynamic role of SIC in the development process of GD and validate personalized iodine guidance (34).

## 7 Conclusions

1. The SIC levels in GD patients are higher than those in the healthy control group, and the SIC levels in untreated GD patients are higher than those in treated GD patients. 2. There is a potential association between SIC and thyroid function indicators. In newly diagnosed GD patients, SIC shows a positive correlation trend with FT4 and TRAb. 3. There are differences in the distribution of patient groups with different iodine contents in the blood, and there are also differences in the levels and positivity rates of TPOAb and TgAb.

## Data Availability

The original contributions presented in the study are included in the article/supplementary material. further inquiries can be directed to the corresponding author.
